# Diet induced weight loss accelerates onset of negative alliesthesia in obese women

**DOI:** 10.1186/1471-2458-5-112

**Published:** 2005-10-18

**Authors:** Patrick Frankham, Caroline Gosselin, Michel Cabanac

**Affiliations:** 1 Département d'Anatomie et Physiologie, Faculté de Médecine, Université Laval, Québec, CA; 2 Groupe Interdisciplinaire de Recherche en Obésité (GIRO), Université Laval, Québec, CA

## Abstract

**Background:**

The physiological and behavioral responses to hypocaloric diet are to increase energy intake to defend a steady body weight. We utilized the method of "negative alliesthesia" for measuring the hedonic reponse to sweet stimulus before (Initial session) and 3 months after entering a weight loss program. The negative alliesthesia test is known by physiologists but few clinical data exist. It is based on the observation that repeated pleasant gustatory stimuli turn into unpleasantness in the process of alliesthesia. At first visit participants repeatedly ingested sweet stimuli until they found them unpleasant and rated quantitatively on a linear analogue scale their hedonic experience. This procedure was repeated every 3 min until participants felt displeasure to end the session. The same protocol was followed after three months of following a weight loss diet. Dieting energy intake was from 1400 – 2000 kcal/d for 8 wk. Energy composition was 50% carb:25% prot: 25% lipid. After 8 wk caloric intake increased by 50 kcal/wk, to reach daily intake of 1800 – 2400 kcal/d. Energy composition was 50% carb:22% prot: 27% lipid. We report results on the effect of slow weight loss on negative alliesthesia in ten obese female participants enrolled in a commercial diet program based on Canada's Food Guide (Mincavi^®^).

**Results:**

Results showed that diet lowered the mean BMI (Initial session 36.8 +/- 1.8 vs. 3 mo 34.9 +/- 1.8 kg/m^2^). At 3 mo the onset of negative alliesthesia, time to abandon experimental session, was shortened (Initial session 33 vs. 3 mo 24 min). The same trend was observed in the time to reach indifference (Initial session 21.9 +/- 3.8 vs. 3 mo 16.2 +/-2.4 min). There was no observed difference in maximum (Initial session +79.5 +/- 11.7; 3 mo +94.5 +/- 9.9 mm) and minimum (Initial session -90.0 +/- 14.4; 3 mo -106 +/- 11.1 mm) hedonic rating.

**Conclusion:**

Earlier onset of negative alliesthesia, as seen in our participants, is not consistent with previous hedonic studies that showed delayed or absent negative alliesthesia in participants when below their initial body weight. Therefore, it is hypothesized that the accelerated onset of negative alliesthesia observed in our obese participants after weight loss is suggestive of a lowered body weight set-point. Factors inherent to the weight loss diet studied here, such as mild energetic restriction, lowered palatability, and diet composition, may have played a role in this experimental outcome.

## Background

Energy balance is dependent upon a constant equilibrium between the energy intake and energy expenditure. The former is controlled by "appetite" through an intermediate phase termed "satiation" and its termination via "satiety" [1-4]. The role of satiety in controlling food intake begins once food has interacted with the receptors on the tongue and nose. The bolus of food follows the oropharyngeal duct to the stomach and duodenum, onward to the jejuno-ileum and colon to be expelled once digested. Along the transit, satiety signals arise from visceral afferent nerve fibers through the gastro-intestinal tract itself and the blood. At every step there is a cascade of neuromodulators that are postulated to participate, in part or entirely, in satiety (bombesin, CCK, PYY, ghrelin, leptin, insulin, glucocorticoids) [5-9]. Maladjustment in any component may favor increase daily intake and thus contribute to an upward drift in body weight and eventually obesity.

Some authors attribute the rising prevalence of obesity [10-13] simply to ever increasing caloric intake [14] and decreased physical activity while others have implicated over- or under-responses of satiating hormones or maladjusted satiety cues [15,16]. Regardless of the underlying etiology of obesity, this pathology is further complicated by such biobehavioral factors as dietary variety, portion size, snacking, cafeteria foods, increased palatability, and low cost of calorie dense foods [14].

In the 50's, Keys' experiment of semi starvation and refeeding in healthy normal-weight men [17] has provided data supporting the existence of autoregulatory feedback signals linking the state of depletion of fat stores to compensatory mechanisms operating via both food intake and regulatory thermogenesis [18-20]. In the following years, a number of authors have defended the hypothesis that body weight is regulated and that there exists a ponderostat [21-45]. According to that point of view, the major signal responsible for the overall stability of body weight is the set-point. That point of view entails that obesity results from a rise in body weight set-point, an analog signal that is the aim the system tends to achieve. If this were not the case, all defense mechanisms would operate to counter the rise of body weight.

Others have argued against the set-point theory and have instead substituted the term settling point [46,47]. According Davis & Wirtshafter [46] the absence of a set-point would then mean the absence of regulation per se. The same point of view against a set-point also existed in temperature regulation. For Webb [48,49] the system defends heat rather than temperature. Such a concept of regulatory biological systems is actually equal to an absence of any regulation and systems operating as simple steady states. With temperature as well as with body weight, the fact that responses (shivering, sweating, alliesthesia, thermogenesis, hoarding etc.) oppose changes to temperature and to body weight minimize the significance of the settling point hypothesis.

In previous studies, our laboratory has found that negative alliesthesia occurred when normal weight subjects were maintained on a ad libitum bland monotonous diet [50]. These subjects also experienced significant weight loss. In addition, when maintained on a bland diet normal weight subjects' lose weight without reporting a sensation of chronic hunger, a pattern understood as a lowering of the body weight set-point [50]. Inversely, when obese subjects lower their body weight while maintained on a caloric restriction diet it has been shown that satiety was decreased or suppressed [51]. The only difference between normal and obese patients is that the response takes place at a higher body weight in the latter [51].

The gustatory pleasure evoked by a sweet stimulus in subjects maintained under varying weight conditions reveals valuable information about the internal state of the biological system [32,51-53]. The decrease of initial pleasure after repeated ingestion of a sweet stimulus demonstrates an important physiological mechanism termed "negative alliesthesia".

The relationship between obese individuals' alliesthesia and their body weight has scarcely been studied in the clinical setting. The aim of the present clinical study was to compare the onset of negative alliesthesia in obese subjects before- and three months after following a weight loss diet.

## Methods

### Study participants

Ten female participants from the greater Quebec City Region were recruited from the Minçavi weight loss program. All participants were at least 18 yr of age and had stable body weight at the time of joining the program. Stable body weight was defined as not having gained or lost more than 2 kg in the past month. Patient demographics are outlined in Table 1. The protocols were described in detail to the participants, without any use of the word set-point, and without mentioning the aim of the study.

**Table 1:** Participant's demographics and descriptive data analyses. Obesity Class I, II & III according to World Health Organization Classification (World Health Organization, Geneva, 1998). Where Class I = BMI 30.0 to 34.9, Class II = BMI 35.0 to 39.9 and Class III BMI > 40.0. At first visit participants chose either Cantin^® ^caramel candy 7-g or Ensure^® ^Vanilla 7-ml as stimulus for determination of hedonic rating. The stimulus was retained for following visits. At first visit (Initial session) participants were asked to subjectively indicate the age at which they considered themselves to have become obese. Our study participants were all female, the age at perceived onset of obesity correlated with first childbearing in all participants.

**Participants Mean (± S.E.) Demographics**	
Sex (n)	
Female	10
Age	31.5 ± 3.2
Body Mass Index †	
Initial BMI	36.8 ± 1,8
3-Month Diet BMI	34.9 ± 1,8
Loss (%)	5.2 ± 0.2
Initial Body Weight (kg)	94.7 ± 3.0
3-Month Body Weight (kg)	89.9 ± 3.0
Loss (%)	5.2 ± 0.2
Obesity Class (BMI)	
I (30.0 to 34.9)	3
II (35.0 to 39.9)	4
III (>40.0)	3
Stimulus chosen	
Liquid (Ensure^® ^liquid diet)	3
Solid (Cantin^® ^caramel candy)	7
Age of Perceived Onset of Important Body Weight ‡	24 ± 2 yr

†: The body mass index is the weight in kilograms divided by the square of the height in meters (kg/m^2^).

‡: This variable is defined as that time when participants were aware that they were "heavier" than "average" individuals. This response was correlated with childbearing in all participants.

Participants were instructed to fast overnight and arrive at our laboratory early the following morning. At each visit, only one participant was taken at a time. Participants completed a general health questionnaire and body weights were recorded before initiating the experimental sessions.

### Inclusion and exclusion criteria

Diabetics (Type I and II) were excluded from participating in the study. Smokers were also excluded because previous experimental results with the same hedonic method used here have shown that transient nicotine can lower the set-point for body weight [54], a result that was confirmed in rats [15].

### Ethics, informed consent and compensation

The study protocol was approved by University Ethics Committee and each participant signed an informed consent before study initiation. Participants received forty dollars per visit as a compensation for their participation in the study. The monetary amount given is believed not to have influenced participant's ratings.

### Minçavi^® ^commercial weight loss program

Minçavi^® ^(phonetically: "thin for life" in French), is a commercial weight loss program, based on Canada's Food Guide recommendations. Upon joining the program, participants, mostly women, receive a recipe book and are explained the importance of eating 3 meals/day and choosing foods from the four major food groups (grain products, fruits/vegetables, milk/dairy products, meat/meat alternate). The proposed recipes are based on inexpensive, readily available whole grain products, vegetables, fruits, lean meats, and low-fat dairy products. Group leaders teach participants how to prepare a variety of nutritious, well balanced and easy to cook meals. Group leaders are people who have lost excess body weight and have been maintaining a healthy body weight for at least two years by following the very program they promote. Participants are free to decide for themselves how much weight they want to lose. However, the group leaders establish the milestones in accordance to a recommended 5% loss in body weight.

Depending on their BMI, sex, age and physical activity, participants are assigned one of the four diet plans that range from 1400 to 2000 kcal/d (5852 to 8360 kJ/d) in the weight loss phase. During that phase, it is estimated that 50% of the energy is derived from carbohydrates, 25% from protein, and 25% from lipids. When participants reach the goal weight, they enter the "weight maintenance phase" and are taught to increase their caloric intake by 50 kcal /wk, in a minimum of 8 wk, to eventually reach a daily intake of 1800 to 2400 kcal/d (7524 to 10 032 kJ/day) depending on energy needs. During this second phase, diet composition changes slightly, with a decrease in protein and an increase in lipid content (carbohydrates = 50%, protein = 22%, lipids = 27%).

In groups of 50 to 100, participants are instructed on how to record everything they consume in a daily journal designed for that purpose and they are invited to share them with their group leader every week. Weigh-in sessions followed by a 30 to 45 min lecture on various topics (e.g., weight loss, nutrition, motivation) and recipe sampling; take place on a weekly basis. In addition, the commercial program provides support from a dietician through telephone line and Internet.

### Sweet stimuli

At first visit, participants were allowed to choose between one of two sweet stimuli that would be presented to them over the experiment. Participants were explained that the stimuli would be ingested repeatedly over the session and retained for the next visit. Stimuli were either Cantin^® ^caramels (7-g) to be chewed or Ensure^® ^Vanilla (7-ml) to be drunk. Two different stimuli were offered, without being tasted, to offer an alternative to those participants who might not like caramel, or would be unable to chew due to dental prosthesis.

### Hedonic ratings

Participants ingested one sweet stimulus, and after 15 s were asked to report their hedonic rating on a linear analogue scale. The entire stimulus was masticated (caramel) and swallowed, or drunk (Ensure^®^). Participants did not rinse after ingesting the stimuli. This procedure was repeated every 3 min until participants felt displeasure and decided to end the experimental session. Participants were instructed that they would have to decide by themselves when they chose to end the session out of displeasure (disgust for the stimulus: negative alliesthesia). Participants were instructed to indicate with a felt pen on a washable board the hedonicity aroused by the stimulus. They were to use an analogue scale with (0) in the middle for an indifferent sensation, a positive (+) for a pleasant sensation, and (-) for a displeasurable sensation. The distance (mm) from zero (0) would indicate the intensity of pleasure or displeasure. After the participant wrote her mark on the scale, the distance from zero (indifference) was measured, then the mark was erased. The repeated ratings were then plotted against time. Subjects performed the first alliesthesia test before starting the diet and the second one, three months after entering the program, while still on the weight loss diet.

### Data analysis

The percent change in body weight at three months from initial session was calculated after the second session. This value was determined by dividing the difference in body weight between experimental sessions by initial session body weight. Value was expressed as a percent. Endpoints measured at each session were:

i. Body Mass Index (BMI). Determined by dividing participant's body weight (kg) / participant's height squared (m^2^).

ii. Time to reach zero rating for the hedonicity of taste sensation. This variable is defined as the time taken by participant to reach indifference after ingesting sweet stimuli every 3 min. This variable is interpreted as indicative of satiation.

iii. Time to abandon experimental session. This variable is interpreted as full satiety through negative alliesthesia since the participant was free to withdraw whenever she chose.

iv. Amplitude of hedonic rating. The highest (maximal) hedonic rating was the most pleasurable rating reported by participants after ingesting sweet stimulus. In opposition, the lowest (minimal) hedonic rating was the most displeasurable rating reported by participants after ingesting a sweet stimulus.

v. To compare rate of alliesthesia, the Kaplan Meier's method was used as for survival rate.

## Results

**Figure 1:**
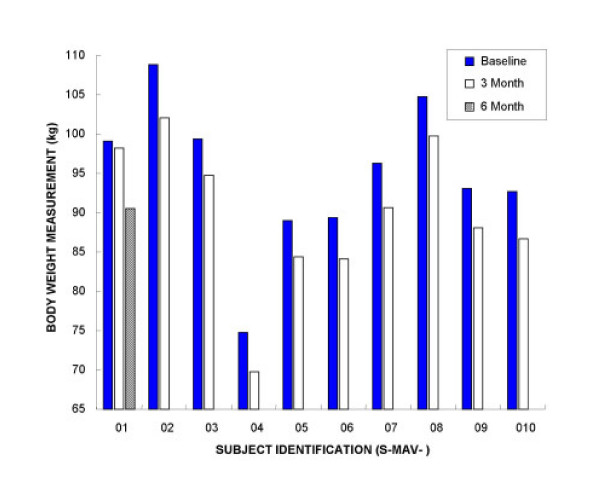
Individual body weight loss in 10 obese participants at Initial session and 3 mo following a commercial regimen (Minçavi^®^). Mean group body weight loss showed a statistically significant decrease (Initial session 94.7 +/- 3.0; 3 mo 89.9 +/- 3.0 kg; Student's paired *t *= 9.90; p < 0.0001; two tailed). The range of body weight loss was from 0.9 kg to 6.8 kg for all participants who followed the Minçavi^® ^diet for three months. One participant (ID. S-MAV-01) was invited to return for a follow-up visit after 6 mo on diet. Her body weight showed a continued decrease of 0.9 kg at 3 mo and 8.6 kg at 6 mo.

After following the Minçavi^® ^weight loss program for three months, mean group body weight was significantly lower (Initial session 94.7 +/- 3.0; 3 mo 89.9 +/- 3.0 kg; Student's paired *t *= 9.90; p < 0.0001; two tailed; Table 1). Maximal weight loss observed in our participants was 6.8 kg and minimal was 0.9 kg (Figure 1). Expressed as BMI, the weight loss program caused a significant decrease after three months (Initial session 36.8 +/- 1.8; 3 mo 34.9 +/- 1.8 kg/m^2^; Student's paired *t *= 10.4; p < 0.0001; two tailed). Body weight loss at 3 mo was 5.2 % ± 0.2 (Table 1)

**Figure 2:**
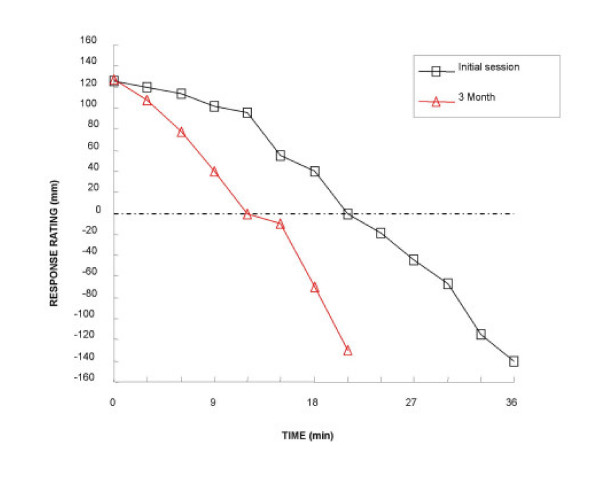
A representative satiation kinetic from one obese participant (Class I) at Initial session and 3 mo following a commercial regimen (Minçavi^®^). The curve shows that time to reach zero rating significantly decreased from 21 min at Initial session to 12 min at 3 mo on diet. The figure also shows that the overall time to abandon the experimental session, negative alliesthesia, was significantly decreased from 36 min at Initial session to 21 min at 3 mo on diet. From this satiation kinetic, the initial rating of pleasure (Initial session 125 mm) remained the same (3 mo 127 mm). The amplitude of rating (minimal and maximal hedonicity) was also similar from Initial session to 3 mo.

**Figure 3:**
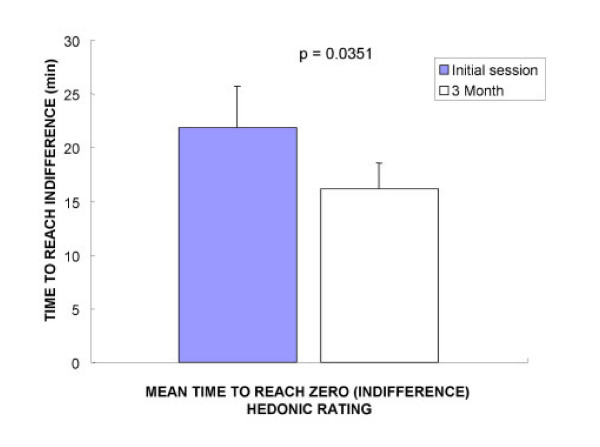
Mean (+/- SE) time to reach zero rating or indifference. The overall time to reach zero rating was significantly decreased after dieting (Initial session 21.9 +/- 3.8; 3 mo 16.2 +/- 2.4 min; Student's paired *t *= 2.48; p = 0.0351; two tailed).

When patients repeatedly ingested sweet stimuli and reported their hedonic rating, the entire cohort of fasted participants started with reports of pleasurable sensation and gave positive hedonic rating. Over the course of the session the positive rating fell to zero or became negative, indicating negative alliesthesia. A representative alliesthesia kinetic from one participant before and while on the Minçavi^® ^diet is shown in Figure 2. The mean time to reach zero rating, or indifference, revealed a statistically significant earlier delay after dieting (Initial session 21.9 +/- 3.8; 3 mo 16.2 +/- 2.4 min; Student's paired *t *= 2.48; p = 0.0351; two tailed, Figure 3). The solid stimulus was chosen by 7/10 of participants, over the liquid stimulus. Regardless of the solid or liquid chosen, participants still yielded identical kinetic profiles for alliesthesia before and after weight loss.

**Figure 4:**
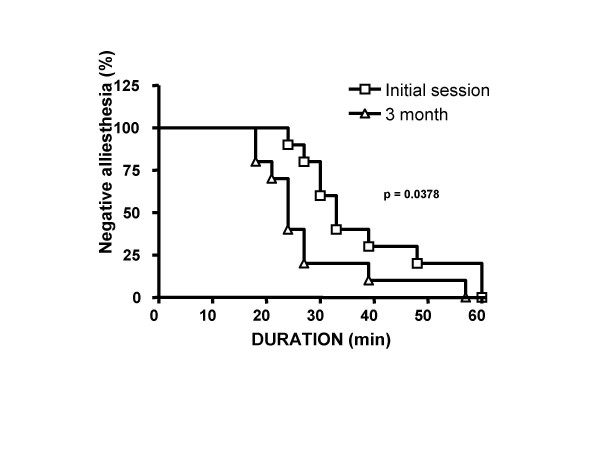
Kaplan-Meier curve was created to determine the time to achieve an event: satiety. Diet showed a significant increase in the satiety rate at 3 mo *vs*. Initial session (Log-rank chi-square = 4.312, df = 1, p = 0.0378). The median time to achieve satiety was lower after diet (24 min vs. 33 min).

In order to follow the time course of alliesthesia in our experimental groups, we created survival curve using the product limit method of Kaplan-Meier. This procedure would cancel the fact that not all participants followed the same time course and stayed for some duration. No event times thus were considered missing or censored. The median time to achieve negative alliesthesia was significantly increased after diet (Log-rank chi-square = 4.312, df = 1, p = 0.0378; Figure 4). Diet lowered the median time to achieve negative alliesthesia (Initial session 33 vs. 3 mo 24 min). There was no difference in maximal and minimal median hedonic rating (maximal Initial session +79.5 +/- 11.7 mm; 3 mo +94.5 +/- 9.9 mm; Wilcoxon = 14.5; p = 0.1934 or minimal Initial session -90.0 +/- 14.4 mm; 3 mo -106 +/- 11.1 mm Wilcoxon = 22.0; p = 0.6250). The absence of detecting a significant difference in this endpoint reveals that participant's hedonic rating for sweet stimulus was unchanged after diet.

## Discussion

Obese participants enrolled in the present study all lost weight by following the Minçavi^® ^weight loss program. Although the overall group weight loss was statistically significant, the clinical relevance of such a loss for any individual participant (range 0.9 – 6.8 kg) cannot be ascertained. However, for obese people even moderate weight loss is thought to be beneficial if not physiologically at least psychologically[55,56]. All but one participant met the objective of a 5% decrease in BMI purported by the Minçavi^® ^program (Figure 1).

Historical data from a controlled clinical trial in morbidly obese participants awaiting bariatric surgery and weight-matched un-operated controls utilizing the same experimental approach over a longer period revealed a similar alliesthesia kinetic [57]. Curve parameters from the present diet study suggest that negative alliesthesia as seen in our participants is comparable to those reported in morbidly obese patients (Historical controls initial session: 25.5 min, vs. Historical controls 3 month: 30 min).

The pleasure aroused by sweet stimuli faded earlier at the three month mark than at the initial session. The relationship of pleasure with intake is obvious as a stimulus that turns toward unpleasantness should be rejected; such negative alliesthesia is suggestive of a satiation process. Earlier onset of negative alliesthesia was observed in each participant who had lost weight. A weak negative correlation was observed when we tested the relationship between amount of weight loss from diet to time to achieve negative alliesthesia (Pearson r = -0.53). Correlation data did however suggest the use of greater patients in future studies utilizing the method. At first visit, all participants yielded a response kinetic, that could be interpreted as three distinct phases, namely; appetite, satiation, and satiety. Because participants all arrived at our laboratory after an overnight fast, their initial rating of hedonicity was positive at every session. Each participant transitioned through to what could be referred to as a "satiation phase", indicative of a zero or indifferent hedonicity rating. The final phase, suggestive of satiety, was the abandonment due to significant displeasure and disgust for the sweet stimulus. Thus, our obese participants displayed a negative alliesthesia pattern similar to that observed in normal weight subjects. It is likely that changes in alliesthesia observed here were linked to that specific diet and that upon returning to their previous dietary habits, subjects would probably return to their baseline response.

Previous experiments have focused mainly on shift in taste preferences in obese and lean individuals after dieting [53,58]. These investigations yielded equivocal results, and assessed the changes in taste correlated with lowered body weight. Our participants did not modify their initial ratings although their weights were lowered from dieting. Such a result would not support a changed taste preference related to body weight in fasted subjects. Cummings, Weigle, Frayo, Breen, Ma, Dellinger, & Purnell, [59] studied the effects of diet-induced weight loss on plasma ghrelin levels. Their subjects showed a significant 17% decrease in BMI after six months. Although the population studied was similar to ours, their experimental approach to weight loss was more drastic than the Minçavi^® ^diet. Subjects from the Cummings study were initially on a three-month liquid diet (1000 kcal/d) and transitioned to a solid diet for the remainder of the study.

## Conclusion

An earlier onset of negative alliesthesia was obvious at three months when participants had lost weight and were still on the Minçavi^® ^diet. In the light of previous work in animals and in humans cited earlier, these findings could be interpreted has a lowering of body weight set-point. Dietary factors may have played a role in this phenomenon. Since Minçavi^® ^promotes a diet that is lower in fat and in added sugar, it is likely that the participants have found it to be less palatable than their usual diet. Earlier work has shown that a decrease in palatability could lower one's body weight set-point [50]. Some studies also suggest that diets that are relatively high in protein [60-62] and that have a low glycemic index [63-67] such as the Minçavi^® ^diet, may promote satiety and weight loss. It is possible such factors, when present in the diet, contribute to lower one's set-point.

Maintaining a lowered set-point, by consuming a sensible diet that promotes satiety and gradual weight loss, may be the key for long-term success, as the body strives to maintain a body weight close to that set-point by reducing food intake and enhancing energy expenditure. Further studies are needed to better understand alliesthesia in obese individuals and its link to body weight set-point.

## Competing interests

The author(s) declare that they have no competing interests.

## Authors' contributions

PF carried out the experimental alliesthesia sessions, and data analyses. MC & CG conceived the study, and participated in its design and coordination and helped to draft the manuscript. All authors read and approved the final manuscript.

**Figure 5:**
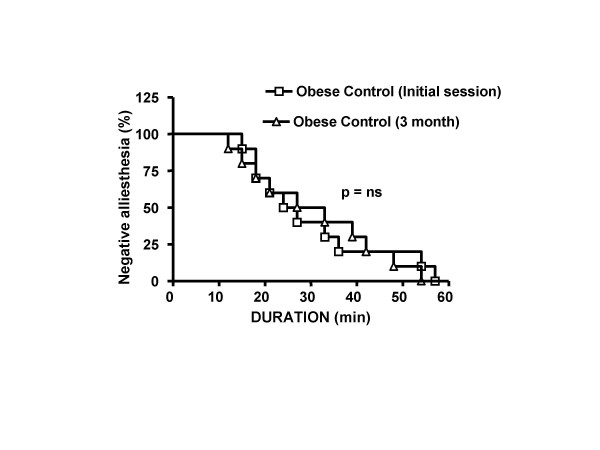
A Kaplan-Meier curve was created using historical control data obtained from morbidly obese patients (BMI > 50) awaiting bariatric surgery. Subjects did not receive any diet intervention. Participants all received the same test for negative alliesthesia described in the methods section. Both groups showed a similar satiety kinetic for sweet stimulus (Log-rank chi-square = 0.045, df = 1, p = 0.8311, NS). The median time to achieve satiety in both groups were comparable (Initial session 25.5 min vs. 3 month 30 min). These results suggest the test is reproducible in a clinical setting for patients.

## Pre-publication history

The pre-publication history for this paper can be accessed here:



## References

[B1] Rolls ET (1981). Central nervous mechanisms related to feeding and appetite. British Medical Bulletin.

[B2] Rolls ET (1984). The neurophysiology of feeding. International Journal of Obesity.

[B3] Blundell JE (1991). Pharmacological approaches to appetite suppression. Trends Pharmacol Sci.

[B4] Blundell JE, King NA (1996). Overconsumption as a cause of weight gain: Behavioural-physiological interactions in the control of food intake (Appetite). Origins and Consequences of Obesity (Series: Ciba Foundation Symposia).

[B5] Woods SC, Seely RJ, Porte DJ, Schwartz MW (1998). Signals that regulate food intake and energy homeostasis. Science.

[B6] Gosselin C, Campfield LH, Cabanac M (2000). Lipostat in the lean rat: evidence for non-causal relationship between glucocorticoids and leptin levels. Appetite.

[B7] McMinn JE, Baskin DG, Schwartz MW (2000). Neuroendocrine mechanisms of regulating food intake and body weight. Obesity Reviews.

[B8] French S, Castiglione K (2002). Recent advances in the physiology of eating. Proceedings of the Nutritional Society.

[B9] Hillebrand JJ, deWied D, Adan RA (2002). Neuropeptides, food intake, and body weight regulation: a hypothalamic focus. Peptides.

[B10] NIH (1998). Clinical guidelines on the identification, evaluation, and treatment of overweight and obesity in adults. NIH publication.

[B11] Taubes G (1998). As obesity rates rise, experts struggle to explain why. Science.

[B12] WHO (1998). Obesity: Preventing and Managing the Global Epidemic.

[B13] Kopelman PG (2000). Obesity as a medical problem. Nature.

[B14] McCrory MA, Suen VM, Roberts SB (2002). Biobehavioral influences on energy intake and adult weight gain. Journal of Nutrition.

[B15] Frankham P, Cabanac M (2003). Nicotine lowers the body-weight set-point in male rats. Appetite.

[B16] Zigman JM, Elmquist JK (2003). Minireview: From anorexia to obesity -the yin and yang of body weight control. Endocrinology.

[B17] Keys A, Brozek J, Henschel A, Mickelsen O, Taylor HL (1950). The biology of human starvation.

[B18] Dulloo AG (1997). Human pattern of hyperphagia and fuel partitioning during weight recovery after starvation: a theory of autoregulation of body composition. Proc Nutr Soc.

[B19] Dulloo AG, Jacquet J, Girardier L (1997). Poststarvation hyperphagia and body fat overshooting in humans : a role for feedback signals from lean and fat tissues. American Journal of Clinical Nutrition.

[B20] Dulloo AG, Jacquet J (1998). Adaptive reduction in basal metabolic rate in response to food deprivation in humans : a role for feedback signals from fat stores. American Journal of Nutrition.

[B21] Yamamoto WS, Brobeck J (1965). Physiological Controls and Regulations..

[B22] Hervey GR (1969). Regulation of energy balance.. Nature.

[B23] Mrosovsky N, Fisher KC (1970). Sliding set-points for body weight in ground squirrels during the hibernation season.. Canadian Journal of Zoology.

[B24] Cabanac M, Duclaux R, Spector NH (1971). Sensory feedbacks in regulation of body weight: is there a ponderostat?. Nature.

[B25] Canguilhem B, Marx C (1973). Regulation of the body weight of the European Hamster during the annual cycle.. Pflüg Arch.

[B26] Keesey RE, Powley TL (1975). Hypothalamic regulation of body weight.. Am Scientist.

[B27] Toates FM (1975). Control Theory in Biology and Experimental Psychology.

[B28] Nicolaïdis S, Meyer P (1977). Physiologie du comportement alimentaire. Physiologie humaine.

[B29] Mrosovsky N, Powley TL (1977). Set-points for body weight and fat. Behav Biol.

[B30] Reddingius J (1980). Control theory and the dynamics of body weight. Physiol Behav.

[B31] Cabanac M, Russek M (1982). Régulation et contrôle en biologie..

[B32] Fantino M (1984). Role of sensory input in the control of food intake. J auton nerv Syst.

[B33] Cabanac M (1985). Influence of food and water deprivation on the behavior of the white rat foraging in a hostile environment. Physiology & Behavior.

[B34] Fantino M, Brinnel H (1986). Body weight set-point changes during the ovarian cycle: experimental study of rats during hoarding behavior. Physiol Behav.

[B35] Keesey RE, Powley TL (1986). The regulation of body weight.. Ann Rev Psychol.

[B36] Toates FM, Evans RAS, Toates FM and Rowland NE (1987). The application of theory, modelling, and simulation to feeding and drinking. Feeding and Drinking.

[B37] Cabanac M, Swiergel AH (1989). Rats eating and hoarding as a function of body weight and cost of foraging. Am J Physiol.

[B38] Mrosovsky N (1990). Rheostasis, the physiology of change.

[B39] Cabanac M, Friedman MI, Tordoff MG and Kare MR (1991). Open-loop methods for studying the ponderostat. Appetite and Nutrition.

[B40] Cabanac M, Richard D (1996). The nature of the ponderostat: Hervey's hypothesis revived. Appetite.

[B41] Flatt JP (1998). What do we most need to learn about food intake regulation?. Obesity Research.

[B42] Jequier E, Tappy L (1999). Regulation of body weight in humans. Physiological Reviews.

[B43] Cabanac M, Michel C, Gosselin C (2000). Corticotropin releasing hormone and body weight regulation: the behavioral approach. Nutrit Neurosci.

[B44] Cabanac M, Russek TM (2000). Regulated biological systems. J biol Systems.

[B45] Cabanac M (2001). Regulation and the ponderostat. International Journal of Obesity.

[B46] Davis JD, Wirtshafter D (1978). Set-points or settling points for body weight?: A reply to Mrosovsky and Powley. Behav Biol.

[B47] Hill JO, Peters JC (1998). Environmental contributions to the obesity epidemic. Science.

[B48] Webb P (1995). The physiology of heat regulation. Am J Physiol.

[B49] Webb P (1997). Continuous measurement of heat loss and heat production and the hypothesis of heat regulation. Ann NY Acad Sci.

[B50] Cabanac M, Rabe EF (1976). Influence of a monotonous food on body weight regulation in humans. Physiol Behav.

[B51] Guy-Grand B, Sitt Y (1976). Origine de l'alliesthésie gustative: effets de charges glucosées ou protido-lipidiques. C R Acad Sci Paris.

[B52] Cabanac M, Pruvost M, Fantino M (1973). Negative alliesthesia for sweet stimuli after varying ingestions of glucose. Physiology & Behavior.

[B53] Esses VM, Herman CP (1984). Palatability of sucrose before and after glucose ingestion in dieters and nondieters. Physiol Behav.

[B54] Cabanac M, Frankham P (2002). Evidence that transient nicotine lowers the body-weight set-point. Physiol Behav.

[B55] Miller-Kovach K, Hermann M, Winnick M (1999). The psychological ramifications of weight management. Journal of Womens Health and Gender Based Medicine.

[B56] Marceau P, Hould FS, Marceau S, Biron S (2001). Malabsorptive obesity surgery. Surg Clin North Amer.

[B57] Marceau P, Cabanac M, Frankham PC, Hould FS, Lebel S, Marceau S, Lescelleur O, Biron S (2005). Accelerated Satiation after duodenal switch. Surgery Obes Relat Dis.

[B58] Herman CP, Polivy J, Esses VM (1987). The illusion of counter-regulation. Appetite.

[B59] Cummings DE, Weigle DS, Frayo RS, Breen PA, Ma MK, Dellinger EP, Purnell JQ (2002). Plasma ghrelin levels after diet-induced weight loss or gastric bypass surgery. New England Journal of medicine.

[B60] Agus MSD, Swain JF, Larson CL, Eckert EA, Ludwig DS (2000). Dietary composition and physiological adaptations to energy restriction. American Journal of Clinical Nutrition.

[B61] Parker B, Noakes M, Luscombe N, Clifton P (2002). Effect of high-protein, high-monosaturated fat weight-loss diet on glycemic control and lipid levels in type 2 diabetes. Diabetes Care.

[B62] Farnsworth E, Luscombe ND, Noakes M, Wittert G, Argyiou E, Clifton PM (2003). Effect of a high-protein, energy restricted diet on body composition, glycemic control, and lipid concentration in overweight and obese hyperinsulinemic men and women. American Journal of Clinical Nutrition.

[B63] Slabber M, Barnard HC, Kuyl JM, Dannhauser A, Schall R (1994). Effect of a low-insulin-response, energy-restricted diet on weight loss and plasma insulin concentrations in hyperinsulinemic obese females. American Journal of Clinical Nutrition.

[B64] Spieth LE, Harnish JD, Lenders CM, Raezer LB, Pereira MA, Hangen SJ, Ludwig DS (2000). A low-glycemic index diet in the treatment of pediatric obesity. Archives of Pediatrics and Adolescent medicine.

[B65] Ball SD, Keller KR, Moyer-Mileur LJ, Ding YW, Donaldson D, Jackson WD (2003). Prolongation of satiety after low versus moderately high glycemic index meals in obese adolescents. Pediatrics.

[B66] Ebbeling CB, Leidig MM, Sinclair KB, Hangen JP, Ludwig DS (2003). A reduced-glycemic load diet in the treatment of adolescent obesity. Archives of Pediatrics and Adolescent Medicine.

[B67] Roberts SB (2003). Glycemic index and satiety. Nutrition in Clinical Care.

